# Adolescents' Mental Well‐Being and Social Support: Mixed Methods Study

**DOI:** 10.1111/inm.70195

**Published:** 2025-12-15

**Authors:** Leona Cilar Budler, Gregor Stiglic, Owen Barr, Majda Pajnkihar

**Affiliations:** ^1^ Faculty of Health Sciences University of Maribor Maribor Slovenia; ^2^ Faculty of Electrical Engineering and Computer Science University of Maribor Maribor Slovenia; ^3^ School of Nursing and Paramedic Science Ulster University Londonderry UK

**Keywords:** mental well‐being, nursing, quality of life, social support, youth

## Abstract

Adolescents are vulnerable to developing mental health problems and mental health disorders if untreated. Various factors can influence their mental well‐being, including personal, interpersonal, community and environmental factors. Interpersonal relations with family and friends may have an influence on the vulnerability of adolescents to developing mental health problems. The aim of this paper is to determine adolescents' mental well‐being and the correlation between adolescents' mental well‐being and the support of family, friends, teachers, and registered nurses. We performed a mixed‐methods study. The survey method was used to collect the data among adolescents, and semi‐structured interviews were conducted among adolescents, parents, teachers, decision makers, and registered nurses. A total of 2972 adolescents participated in the quantitative part of the study. The mental well‐being of adolescents is positively correlated (*r* = 0.624) with their social support, while their mental well‐being (*r* = −0.286) and social support (*r* = −0.239) decline with age. Furthermore, the perceived level of support from registered nurses did not differ significantly by age (*r*(2965) = −0.004, *p* = 0.863) or gender (*W* = 310 616, *p* = 0.903). Qualitative findings further emphasised that adolescents perceive registered nurses as important sources of advice, guidance, and emotional support, underscoring the nursing contribution to adolescent mental health promotion. The mental well‐being of adolescents is related to their interactions and interpersonal relations with parents, friends, and teachers. In ensuring the mental well‐being of adolescents, we must consider the multidimensional model of well‐being. Adolescents' mental well‐being declines with age and is higher among adolescents who receive more social support. Therefore, future interventions and actions should focus on social support and interdisciplinary work.

## 1 Introduction

In recent years, researchers have been discussing the relevance of the definition of mental health regarding positive aspects (Galderisi et al. 2015) and the adequacy of those for different age groups. Lack of conceptual clarity is evident in mental well‐being research (Baldwin et al. 2021) because there is no universal definition of mental well‐being. However, MacKean (2011) discussed mental well‐being as a state of positive psychological and emotional health. Mental well‐being differs among different nations, genders, and age groups. It can be deteriorated by various stressors brought on by growing up. Adolescence (the stage between 10 and 19 years) (World Health Organization 2014) is a period of life characterised by heightened sensitivity to social stimuli and social interaction (DPhill et al. 2020). The concept of social support refers to the salutary content of interpersonal relations. It is a predictive factor of mental health status (Turner and Turner 2013). Also, it is known that a lack of social support, social isolation or dysfunctional social relationships contributes to the development of various mental health problems in children and adolescents (Thompson et al. 2015). Thus, the proposed study aims to investigate if support from family, friends, teachers and registered nurses correlates with adolescents' mental well‐being.

### 1.1 Background

The Government Office for Science in the UK (2008, 42) defined mental well‐being as ‘a state in which the individual can develop his/her potential, work productively and creatively, build strong and positive relationships with others, and contribute to his/her community’ Stewart‐Brown and Janmohamed (2008) state that mental well‐being covers two perspectives: the hedonic, the subjective experience of happiness and life satisfaction, and the eudemonic perspective, the positive psychological functioning, good relationships with others, and self‐realisation. Sarriera and Bedin (2017) proposed a multidimensional approach to well‐being that combines different aspects of well‐being: psychological well‐being (self‐concept, life purpose, spirituality), subjective well‐being (positive effects, adverse effects, life satisfaction, emotional intelligence), socio‐community well‐being (human rights, sense of community, environmental satisfaction) and psychosocial well‐being (interpersonal relationships, leisure, technology use).

The critical factors in protecting adolescents' mental well‐being are supportive parenting, secure home life, and a positive school environment (Wille et al. 2008). It has been discussed that social support promotes general health, quality of life, and well‐being (Stewart and Suldo 2011). Regardless of the definition and form of it, a family must present a sense of belonging, bonding, closeness, and attachment to each other (American Psychological Association 2002). Stewart and Suldo (2011) found that parents' support is a significant predictor of adolescents' mental well‐being. Rothon et al. (2012) found that good paternal and maternal relationships, high parental surveillance, and frequency of evening meals with family were associated with lower odds of poor mental health in adolescents. The relationship quality between the child and parents impacts the child's empathic abilities in adolescence (Boele et al. 2019).

To establish independence, adolescents often orient themselves toward their peers and friends (American Psychological Association 2002). Positive relationships with friends are linked with better adolescents' mental well‐being (Moore et al. 2018). In a study in 2014, adolescents in Slovenia rated their friends' support as average (Pucelj et al. 2016). Those who perceive greater support from classmates experienced fewer symptoms of internalising distress (Stewart and Suldo 2011). In the meta‐analysis, authors Boele et al. (2019) found that adolescents with higher‐quality relationships with peers show more concern and understanding for others than those with lower‐quality relationships.

For many adolescents, school is a prominent part of their lives. In school, they develop relationships with peers and numerous cognitive skills (Kiuru et al. 2020). Support from teachers was found to influence adolescents' self‐esteem and mental well‐being (Moore et al. 2018). There is strong evidence that suggests the implementation of interventions to teach social and emotional skills at school. Those interventions have a positive impact on adolescents' attitudes toward themselves, others, and school, as well as commitment to the school and academic performance (World Health Organization 2018). On the other hand, teachers report that they do not have sufficient knowledge, experience, and training for supporting adolescents' mental health needs. They perceive themselves to be responsible for implementing school‐based interventions but believe that school psychologists have a greater role in adolescents' mental well‐being support (Reinke et al. 2011). The priority for schools must be given to teacher training and professional development in the field of adolescents' mental well‐being support (Graham et al. 2011). A mental health curriculum framework should be implemented for the teachers' training and preparation to ensure effective support for adolescents' mental health and well‐being (Kratt 2018).

For years, school nurses have been integral to the educational system, providing not only general health care but also mental health promotion and prevention for children and adolescents (Houlahan 2018). Unlike mental health physicians, who primarily focus on diagnosis and treatment, nurses are consistently present in school settings, allowing them to establish rapport and trust with students. This accessibility positions nurses as approachable and trusted figures, capable of identifying early signs of mental health issues and providing timely intervention and support. Despite some inconsistencies in nursing education, research indicates that school nurses who receive targeted mental health training can effectively deliver psychosocial support and contribute to the overall quality of care (Ravenna and Cleaver 2016). McGorry et al. (2007) emphasise the importance of early detection of mental health problems to mitigate the risk of long‐term disorders. Nurses, due to their ongoing presence and established relationships within schools, are well‐positioned to act as frontline mental health promoters, bridging the gap between students and specialised mental health services (Torkar et al. 2013). Thus, the focus on nurses in this study is not only justified by their accessibility and educational role but also by their unique ability to engage with adolescents in preventive care and ongoing mental health support, a role that is less feasible for mental health physicians who are typically less present in the school setting.

The proposed study contributes to existing literature by going beyond previous research in several important ways. First, it employs a mixed‐methods design that integrates validated quantitative scales with grounded theory‐based qualitative analysis. Second, it directly compares primary and secondary school adolescents, thereby capturing developmental differences in perceived social support and mental well‐being. Third, while prior studies have primarily focused on individual groups such as family, friends, or teachers, this study examines all population groups simultaneously, with particular attention to the role of registered nurses. Fourth, the study includes a relatively large sample of adolescents (*N* = 2972) in the quantitative part, enhancing the generalisability of findings. Fifth, it incorporates five focus groups with adolescents, parents, teachers, registered nurses, and decision makers, allowing for a deeper understanding and triangulation of quantitative results. Finally, this study is among the first to explore the state of adolescent mental well‐being in Slovenia, where such research has been lacking, and to investigate the association between mental well‐being and social support, a link that has received limited attention both nationally and internationally.

## 2 Methods

### 2.1 Aim

The aim of this study was to determine adolescents' mental well‐being and the correlation of adolescents' mental well‐being with the support of family, friends, teachers, and registered nurses. We explored the role of the registered nurse in maintaining adolescents' mental well‐being.

### 2.2 Study Design

We used a mixed‐method approach that combines quantitative and qualitative methodology. Using quantitative methods, we gained insight into the state of adolescents' mental well‐being and support from parents, friends, and teachers. Using qualitative methods, we assessed adolescents' perceptions of mental well‐being and support from adolescents, parents, teachers, registered nurses, and decision makers.

### 2.3 Setting, Participants, and Procedures

The main criterion for inclusion in the quantitative part of the study was the adolescent age between 10 and 19 (World Health Organization 2014). School‐based random sampling included all available adolescents from 22 primary schools (5.0% of all elementary schools) and 12 secondary schools (5.0% of all secondary schools) in Slovenia. The minimal required sample size was calculated based on data from the Statistical Office of the Republic of Slovenia (2017). The envisaged final number of participants involved was determined by the size of the total population of adolescents, degree of confidence, and margin of error (Qualtrics 2018), which amounted to 384 adolescents. The sample size was increased to avoid the risk of attrition and dropout during the study. The response rate during the study was 42.6.

In the Grounded Theory Method, theoretical sampling was used to gain a broad view of the concept of interest groups studied. The sample was selected based on the knowledge of adolescents' mental well‐being to get the most usable information about adolescents' mental well‐being in correlation with social support. Primary and secondary schools that were involved in the quantitative part of the study were asked to participate in the qualitative part of the study. The recommended focus group size is six to ten participants (Klemenčič and Hlebec 2007), so we assigned eight participants to each focus group. Focus groups were conducted among sixteen adolescents (eight primary school students and eight secondary school students), eight parents, six teachers, six RNs, and three legislators who gave consent to participate in the survey. Adolescents were individuals between 10 and 19 years of age (WHO 2014). Parents of children aged between 10 and 19 were also involved, as well as teachers in primary and secondary schools. RNs and legislators were also involved. Representatives from the Ministry of Health of the Republic of Slovenia, the Chamber of Nursing and Midwifery of Slovenia, and representatives of the Extended Advisory Board for Nursing care with the Ministry of Health were involved. Recruitment in focus groups was based on theoretical sampling. There were only three legislators who agreed to participate in the focus group.

### 2.4 Research Instruments

The KIDSCREEN‐27 measures physical well‐being, mental well‐being, autonomy, parental relationships, support and peer support, and the school environment (Ravens‐Sieberer, et al. 2007). It consists of five subscales: physical activity and health, general well‐being, and emotions about oneself, family and leisure, friends, school, and learning. Each item was scored on a five‐point Likert scale ranging from 1, meaning ‘not at all’, to 5, meaning ‘very much’. The KIDSCREEN‐27 was validated in a pilot study among adolescents attending primary and secondary schools in Slovenia using a six‐step analysis (Dima 2018). It showed good psychometric properties and is suitable for use among adolescents in Slovenia to obtain more information about social support. Specifically, the overall KIDSCREEN‐27 demonstrated excellent reliability with a Cronbach's alpha of 0.93 and an omega coefficient of 0.93.

The Warwick‐Edinburgh Mental Well‐being Scale (WEMWBS) was developed in Scotland in 2006. The scale includes 14 items and measures positive mental health and mental well‐being over the past two weeks. When choosing the answers, a five‐point Likert scale is offered and ranges from ‘none of the time’ to ‘all of the time’. This study was registered on the official website before the use of the scale. A translated scale was used (Cilar 2017), which followed the authors' guidelines for the translation process of the scale. A sum of all answers results in a total WEMWBS score, which can be interpreted as poor (scores between 14 and 41), moderate (scores between 42 and 59), and excellent (scores above 60). The minimal score in WEMWBS is 14, with a maximal possible score of 70. The WEMWBS scale demonstrated strong psychometric properties, with a Cronbach's alpha of 0.89 and an omega coefficient of 0.89.

In the qualitative part of the research, semi‐structured interview guides for conducting focus groups were developed after the quantitative part of the research.

### 2.5 Data Collection

Data collection in the quantitative part of the research took place in the second half of 2019. Certain items (1, 9, 10 and 11) in the KIDSCREEN‐27 scale were reversed when scoring the scale as suggested by the authors. The total score was calculated by summing up all the responses. Higher scores indicated better quality of life. Additional parts (Healthcare and Demographic Data) were added to explore sociodemographic variables in correlation with adolescents' mental well‐being. Permission to use both scales was obtained from authors prior to the start of data collection. Printed consent forms, sheets of additional information, and questionnaires were delivered to each school. After collecting the informed consents, teachers distributed the questionnaires. The adolescents completed the questionnaires in a written (printed) version. The completion of the questionnaires lasted approximately 15–20 min. The data were analysed using R v 3.6.1. programming language for statistical analysis (R Development Core Team 2005).

In the qualitative part of the research, we used the guide for a semi‐structured interview with primary and secondary school adolescents, parents, teachers, registered nurses, and decision makers. It is important to note that the study did not assess whether students received formal mental health care or structured educational sessions specifically provided by nurses. We conducted interviews with one focus group per week, as the grounded theory method requires that sampling, data collection, and data analysis take place simultaneously and involve continuous comparative analysis. In adolescents younger than 16 years, parents completed the consent form. Data collection lasted from September to November 2020 in selected primary and secondary schools in Slovenia. Each interview with a focus group lasted 60 min. The audio recording of the interviews was performed with the prior approval of the institutions and the consent of the participants. Steps by Corbin and Strauss (2008) were followed in developing the grounded theory: theoretical sampling, data collection and analysis, open coding, constant comparison, axial coding, selective coding, and integration. Sampling, collection, and analysis of data using the grounded theory method take place in constant interaction and include a comparative analysis.

### 2.6 Data Analysis

In the quantitative part of the research, the survey method was used to collect data. Data analysis involved the use of descriptive and inferential statistics (Polit and Beck 2017). Questionnaires, where more than 50.0% of the data was missing, were removed from further analysis. In other cases where less than 50.0% of the data was missing, data was imputed using the missForest function (Stekhoven 2016). Psychometric testing of both scales was performed to ensure the validity of the instruments following the six‐step protocol proposed by Dima (2018). An open‐ended question was analysed using a Latent Dirichlet Allocation (LDA) (Pietsch and Lessmann 2018). Semantic analysis was conducted using an RQODA package (Huang 2014).

Analysis of the qualitative data followed the grounded theory approach by Corbin and Strauss (2008). The process included line‐by‐line open coding, axial coding, and selective coding, carried out iteratively with constant comparison. In open coding, initial codes were developed from repeated readings of transcripts and organised into subcategories through processes of reviewing, comparing, and categorising. Axial coding involved linking codes and categories within a paradigm model that encompassed causal conditions, context, actions/interactions, and consequences. During selective coding, a central category was identified and integrated with other categories to form a conceptual framework. Throughout the analysis, memos were used to trace coding decisions and theoretical insights, while field notes informed the interpretation of interactions during the focus groups. The end result was a conceptual description that synthesised categories into theoretical propositions about adolescent mental well‐being and social support.

## 3 Results

A total of 2972 adolescents participated in the study. Demographic characteristics are presented in Table 1. A total of 768 (54.6%) females in primary and 969 (66.6%) in secondary school, and 638 (45.4%) males in primary and 487 (33.4%) in secondary school participated in the study. The mean age among adolescents in primary school was 12.2 (SD = 1.5) and 16.4 (SD = 1.3) among adolescents in secondary school.

**TABLE 1:** Sample characteristics.

	Primary school adolescents	Secondary school adolescents
	(*n* = 1489)	(*n* = 1483)
	***M* (SD)**	***M* (SD)**
Age	12.2 (1.5)	16.4 (1.3)
Gender	***n* (%)**	***n* (%)**
Female	768 (51.6%)	911 (66.8%)
Male	721 (48.4%)	452 (33.2%)
School year	***n* (%)**	***n* (%)**
1st	/	400 (29.3%)
2nd	/	381 (28%)
3rd	/	252 (18.5%)
4th	/	311 (22.8%)
5th	264 (17.7%)	19 (1.4%)
6th	305 (20.5%)	/
7th	266 (17.9%)	/
8th	277 (18.6%)	/
9th	377 (25.3%)	/
School programme		
General	/	504 (34.7%)
Medical	/	274 (18.9%)
Touristic	/	103 (7.1%)
Educational	/	194 (13.4%)
Computer sciences	/	52 (3.6%)
Chemistry	/	224 (15.4%)
Other	/	102 (7.0%)

Abbreviation: *n* = number.

**FIGURE 1:**
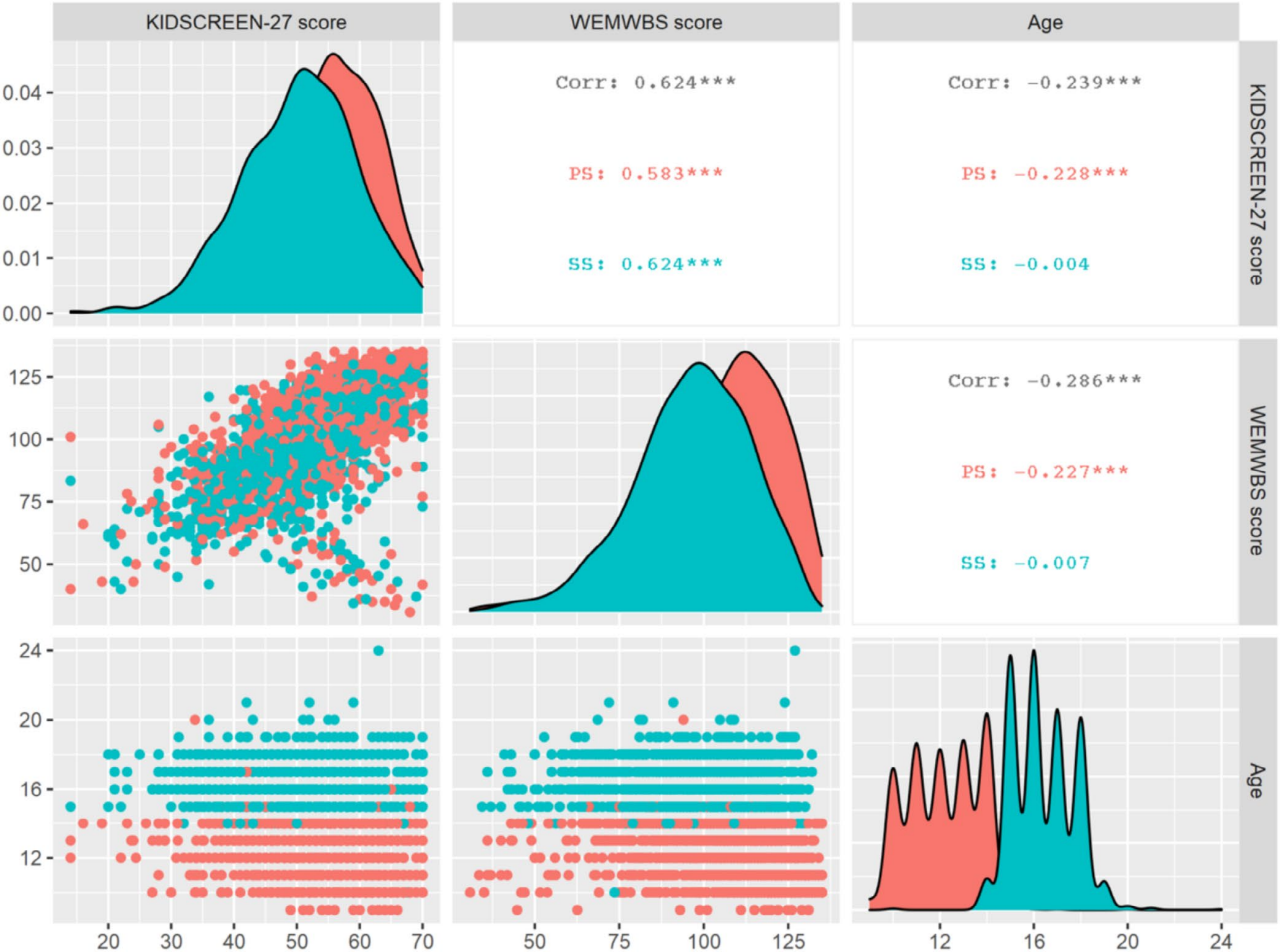
Correlations between age, social support and mental well‐being. *Note*: *** = *p* < 0,001.

Correlations between age and school were checked for both scales (Figure 1).

Adolescents' mental well‐being (*r* = −0.286) and quality of life decrease (*r* = −0.239) with age. Adolescents' mental well‐being is positively correlated (*r* = 0.624) with their quality of life.

**FIGURE 2:**
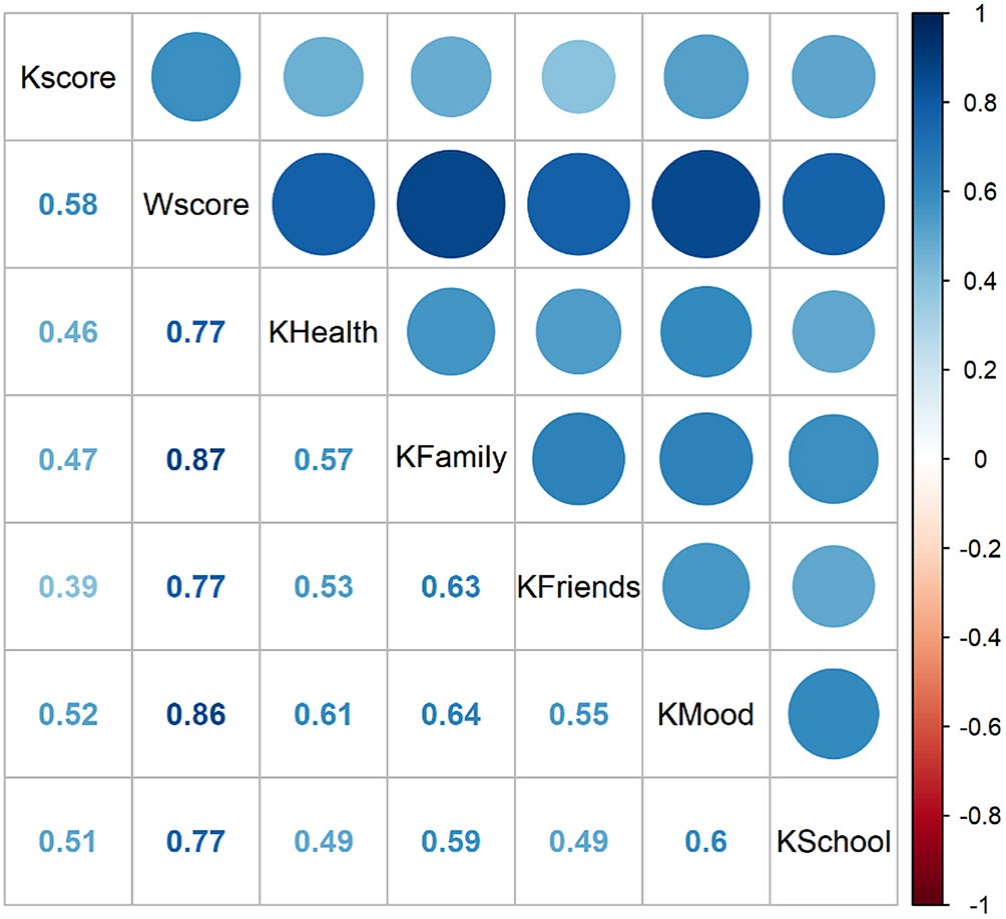
Correlations between social support and mental well‐being of primary school adolescents.

**FIGURE 3:**
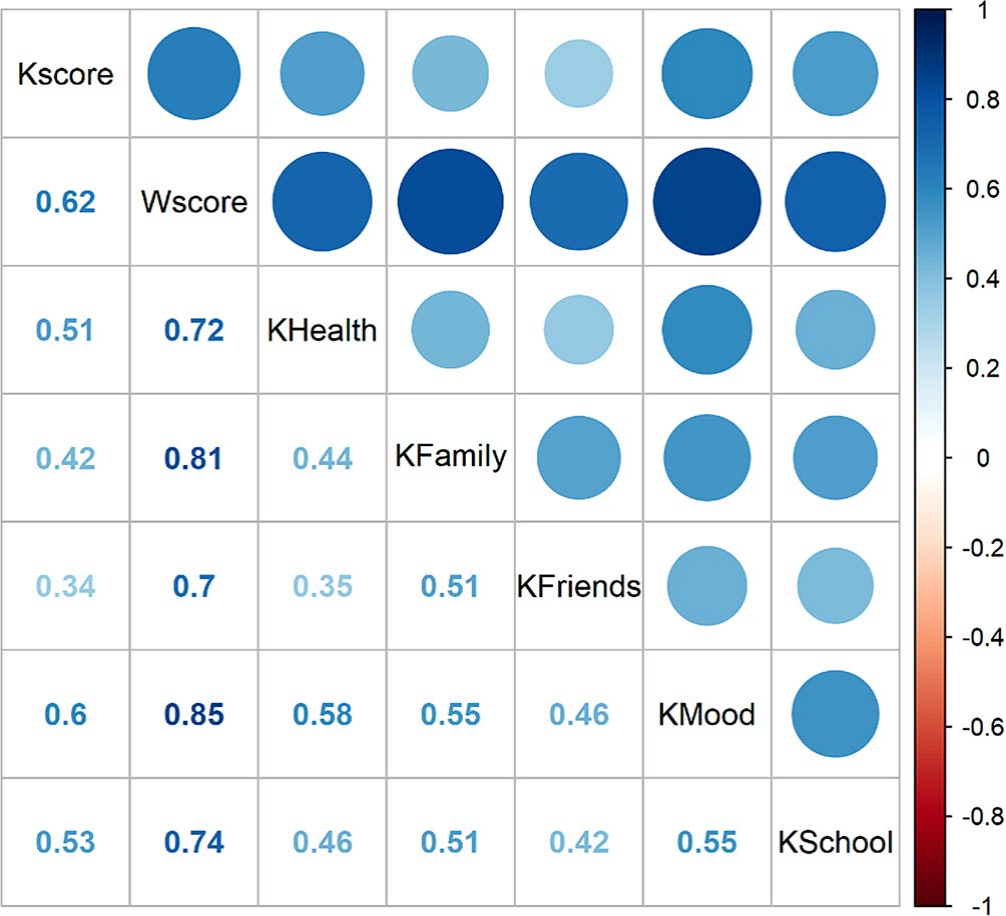
Correlations between social support and mental well‐being of secondary school adolescents.

Correlations between items among KIDSCREEN‐27 subscales are positive among both primary school adolescents (Figure 2) and secondary school adolescents (Figure 3).

In the quantitative analysis, we found a significant positive correlation between social support and adolescents' mental well‐being (*r* = 0.624, *p* < 0.001). Support for adolescents by their family is positively related to their mental well‐being (*r*(2965) = 0.469, *p* < 0.05). Furthermore, support for adolescents by friends is positively related to their mental well‐being (*r*(2965) = 0.373, *p* < 0.05). Finally, the perceived level of support from the registered nurses does not differ between students by age (*r*(2965) = −0.004, *p* = 0.863) and gender (*W* = 310 616, *p* = 0.903).

The quantitative data also revealed that perceived social support from family, friends, and teachers decreased with age, while mental well‐being scores also declined as adolescents progressed through school levels.

Focus group questions were developed based on the quantitative part of the results. The core category was named ‘Adolescents' interpersonal therapeutic relationship’.

### 3.1 Understanding of Adolescents' Mental Well‐Being

All focus group participants were asked about their opinion on adolescents' mental well‐being. Primary school students have had problems understanding the term. They described *mental health problems* as associated with mental disorders:

Yeah, that's how … how you're in the bad mood if you're depressed. (primary school adolescent 1)

At the end of the discussion, they were asked again about mental well‐being. Primary school students perceive mental well‐being as *a way of living and thinking, and an absence of bad relations*.

Primary school adolescent 2 stated: ‘Our thinking’.

Primary school adolescent 4 added: ‘Absence of quarrels. If you don't fight’.

Primary school adolescent 7: ‘Worldview’.

Primary school adolescent 4: ‘How do you live, what motivates you, if anything motivates you’.

Secondary school students stated that mental well‐being is a *positive term* that is closely related to mental health, everyday well‐being, reaction to certain situations, freedom to enjoy certain situations and self‐image. Secondary school student 1 described mental well‐being as:

Basically … we are all energies, the body is itself … the body, but yes basically yes, we work with it ourselves, otherwise we are all energies. Everyone has their own energy.

Registered nurses stated that adolescents' mental well‐being is a positive term correlated with their living environment and social support:

…I think mainly that they live in one secure, safe environment, home environment, that they are accepted as they are and that they can express their opinion without condemnation without anyone to point their finger at them, humiliate them, that they are accepted among their peers, that there is no peer violence (uhm) and above all that they get along with their parents because it all comes from the family. (registered nurse 1)

Registered nurse 6 agreed and added:

That you have a good self‐image. To feel good (noise) in your skin.

A teacher agreed with the view of adolescents' mental well‐being as a positive term and added:

Yeah, to me that seems like a positive self‐image. It seems to me that a child has empathy for others.

Decision makers were asked the same. Their opinion is that the adolescent's mental well‐being is a positive term correlated with mental health and mental disorders. They also added:

That is, I would say that it is basically primarily one positive dimension. That is, some characteristics, the ability of children and adolescents to function in society and in themselves emotionally to exploit their potentials. They are associated with mental health or mental disorders, but it may be a separate concept that needs to be addressed and understood differently than what we have different in focus when talking about the social health of children and adolescents… (Decision maker 2)

### 3.2 Adolescents' Mental Well‐Being Correlation With Interpersonal Relations

In focus groups, both primary and secondary school students were asked about important people in their lives when assuring mental well‐being. Primary school students stated that the most important relationship is the relationship with *family members* (especially the mother).

Registered nurses agreed that the biggest influence is the *family*. Moreover, *classmates* and *friends* have a big role in adolescents' mental well‐being:

The first is definitely the family, isn't it? So … the wider environment, the classmates. (Registered nurse 2)

Primary school students also added that *friends* are the ones they trust and talk to about problems:

I usually talk to a friend first. And even if we all wrote the tests poorly, we know that it will be easier for my mother to understand, but if I write poorly, she is a little angrier. (Primary school adolescent 1)

On the other hand, secondary school students pointed out that family has an impact, but not as big as *friends*:

But since family is only biologically connected, it's nothing, it's literally nothing, does not mean anything. I have a friend, two friends, that's how it is for me. (Secondary school adolescent 2)

In relation to adolescents' mental well‐being impacted by social support, secondary school adolescent 6 stated that an adolescent has to process problems with oneself first:

It's best to deal with yourself first. To be aware of yourself. Basically, by being aware of your acts. You have to process it yourself.

From the parents' perspective, *adolescents find friends more important than family members*:

I think it's primary family, no, just (uhm) like the lady said, no, I think now the kids are at such age when (uhm) we aren't (uhm) … from their point of view we are not in the first place for these children. (Parent 4)

When talking about good interpersonal relations, secondary school students stated that the relationship should be based on the following *principles of good interpersonal relations*: good communication, trust, openness, understanding, support, patience, awareness of differences, positive opinion, honesty, equality, and respect.

As described, adolescents' mental well‐being is closely related to their relations with others. Registered nurse 3 pointed out the role of the family:

Absolutely, the family is the one that is the first cell and is involved and we missed a lot, and if we don't start working on it, in kindergartens, as we said before, we have missed generation.

Registered nurse 1 stated that *teachers* have a small role in providing adolescents with mental well‐being, as they do not have much autonomy. Registered nurse 1 also stated:

Don't get involved in their work, because they went through education anyway, they have the knowledge, they gain experience with mileage, but more autonomy. Don't get involved in children's education.

Registered nurses think that *people, in general, can talk to nurses more easily than to doctors*, which may be the reason why adolescents perceive registered nurses as important persons in their life:

It's easier to tell my nurse there, ‘I haven't gone to… I don't know…to poop for three days than to go to the doctor if he doesn't even look me in the eye, for example. I don't know. The sister is always that confidant. (Registered nurse 6)

### 3.3 Factors and People That Influence Adolescents' Mental Well‐Being

Registered nurses emphasised that to maintain an adolescent's mental well‐being, it is important to provide a *safe living environment*:

…basically, I think mainly that they live in one secure, safe environment, home environment… (Registered nurse 1)

Also, registered nurse 1 added that mental well‐being is closely related to *good interpersonal relations and a safe environment in which adolescents can express themselves and are accepted by others*. Registered nurse 3 stated that an adolescent's mental well‐being is when:

an individual is capable of accepting criticism, that an individual is capable of cooperating with others … (uhm) That an individual is also capable of (uhm) waiting a little, no, for one thing. (Uhm) Yes … I will say that he also has confidence in himself, but yes, that these are not empty words, which for the most part is now all seemingly full of self‐confidence, but in reality, they are very bad.

Registered nurse 2 agreed with others and pointed out that *the generation has changed, as well as adolescents' values*:

I agree with you here, but you know what I'm looking at here, no, because unfortunately, we notice something, no, that (uhm) there are practically no more of those values, some morals, no, some ethics, no. And (uhm) young people too little …

Parents think that *if they communicate with their child about the positive and negative consequences, the adolescent can decide on their own if and how they will use social media*. They also added that modern technology is one way of relaxing for adolescents:

It's not from us because we didn't live that way, but today's world is set that way and it's just maybe, it's really a child's relaxation, right? That maybe that time he is disconnected, but he's in some unreal world, no, just those games, no, but he says to himself, here I do the way I want, no. There are no external influences here. (Parent 4)

Also, parents think that *teachers expect too much from children* and that both *parents and adolescents do not get the appropriate and needed support* from teachers:

My son had health issues (uhm) in third grade he got an epileptic seizure. And let's say here in the school I didn't get any support in the sense that it would be easier for him. It was harder for him. (Parent 5)

Registered nurses were asked about their role in supporting adolescents with mental well‐being. Registered nurse 1said:

In general, I will say from the first visits to the counselling centre onwards, no, as far as talking to parents is concerned, no, health education work absolutely (uhm).

### 3.4 The Role of Registered Nurses in Providing Adolescents' Mental Well‐Being

Primary school students stated that they meet with a nurse only *when they are sick* or when performing *systematic health visits* in school. Primary school students gave short answers; thus, some answers are just listed and not cited. Results are similar to answers in the quantitative part of the research. Students stated that nurses *talk with students*, *help*, *care*, *calm down*, *understand*, *teach* and *advise*, give hope, and *speak positively*. Moreover, they agreed that *it is easier to talk to a nurse than to a doctor*.

Secondary school students agreed that nurses have a significant role *when more serious mental health problems occur*:

I lean more towards friends. She just doesn't have any…unless it's something more serious. (Secondary school adolescent 1)

They also emphasised that a nurse *has the needed knowledge* and that in certain situations *it is easier to talk to a nurse than to a friend*:

But sometimes it's also easier because she can't criticise you, because she doesn't know you. (Secondary school adolescent 3)

Secondary school adolescent 2 added that:

…it just depends on the situation. If it is more serious, the point is that you need someone who knows how to deal with it and knows how to help you.

On the other hand, secondary school adolescent 5 states:

Maybe it is worse, because you're ashamed to talk to someone you don't know.

Primary and secondary school students perceive registered nurses as important people in maintaining their mental well‐being. Registered nurses' roles are different: a foreign person, a source of information, a teacher, a leader, a counsellor, and an expert. Registered nurses' role is also therapeutic as they perform individual talks with adolescents, help them, advise them, and perform other activities to support their mental well‐being. Identified roles are in accordance with nurses' roles described by Peplau (1952, 43–72).

Teachers agreed that registered nurses do not spend much time in schools; thus, their role is not significant:

They, for example, had a systematic health visit this year. Well, they have those conversations systematically… on certain topics, but there is certainly not enough of that… that this would have an impact on them. (T2)

In the qualitative findings, adolescents consistently emphasised the importance of peer support and the perceived decline in family support as they aged, aligning with the quantitative results indicating that social support decreases with age. Additionally, while quantitative data showed a positive correlation between overall social support and mental well‐being, qualitative data provided more nuanced insights into the nature of these relationships. For instance, primary school students highlighted the importance of family as the primary source of support, whereas secondary school students identified friends as the most influential support figures. Furthermore, the qualitative findings highlighted that although students perceived registered nurses as knowledgeable about health and approachable, these interactions were informal and unstructured. This contrasts with the quantitative data, which did not specifically measure interactions with registered nurses but did suggest a general decline in perceived social support over time. Thus, while the quantitative data indicated a broad trend of decreasing support with age, the qualitative findings provided specific contextual examples and highlighted the absence of structured support from registered nurses.

## 4 Discussion

The integration of quantitative and qualitative data provides a comprehensive understanding of the patterns of social support and mental well‐being among adolescents. The quantitative findings revealed a significant positive correlation between social support and mental well‐being, consistent with prior research (Sarriera and Bedin 2017; Ravens‐Sieberer et al. 2014). However, the qualitative data elaborated on the specific nature of these relationships, emphasising how support from family, friends, and teachers evolves over time and is perceived differently by primary and secondary school students. While the quantitative data identified an overall decline in perceived social support and mental well‐being with age, the qualitative data revealed that this decline is particularly pronounced in the transition from primary to secondary school, where peer support becomes more influential and family support diminishes. Additionally, the role of registered nurses, though not directly measured in the quantitative analysis, emerged as a significant theme in the qualitative findings. Participants acknowledged the potential role of nurses in providing mental health support but noted that these interactions were typically informal and unstructured. This qualitative insight highlights a potential area for intervention that was not captured in the quantitative data, suggesting that structured mental health education programs delivered by registered nurses could address the gap in perceived social support, particularly in secondary school settings.

**FIGURE 4:**
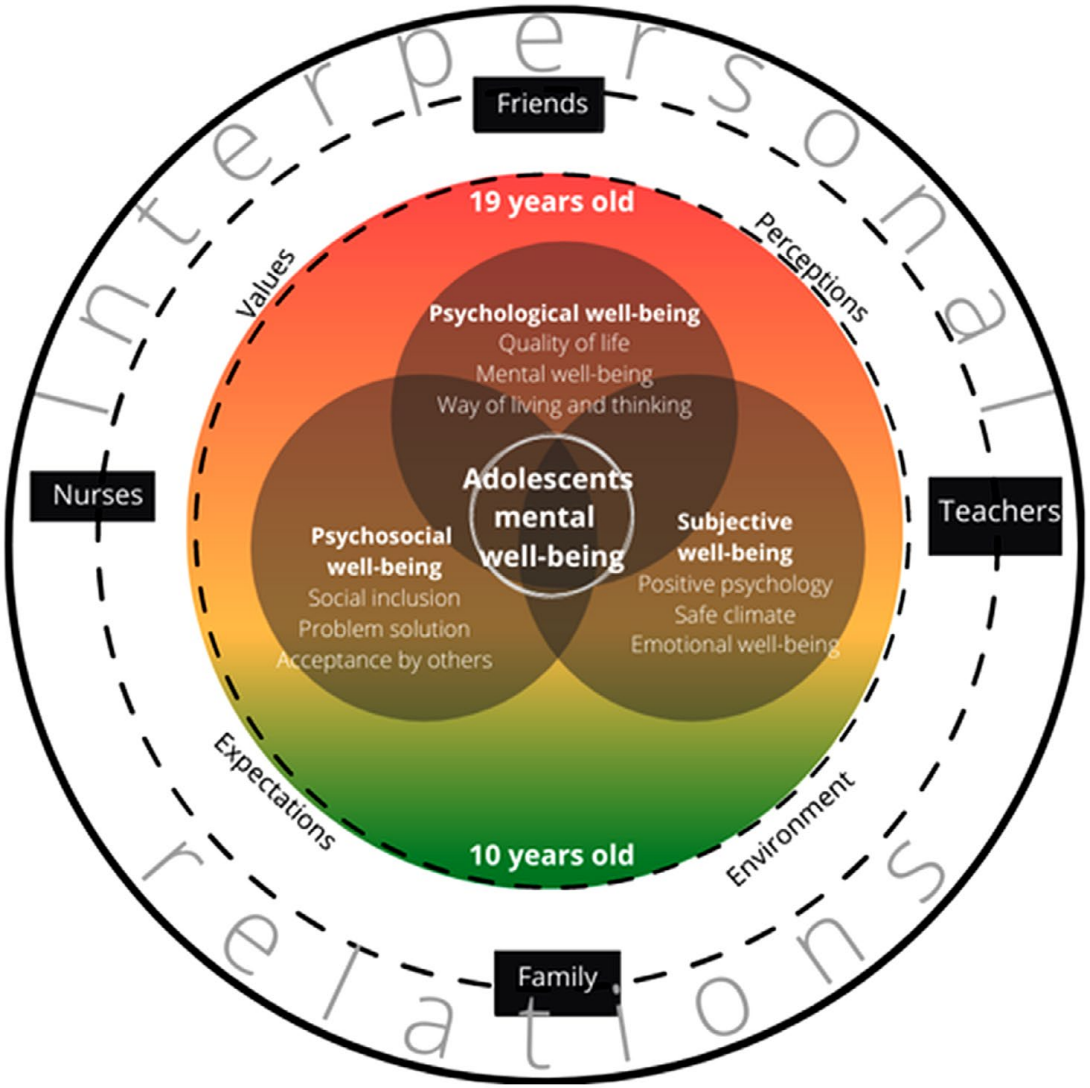
Interpersonal relationship model.

A theoretical model for adolescents' interpersonal relations was developed considering the theory of interpersonal relations (Peplau 1952) and the multidimensional well‐being model (Sarriera and Bedin 2017) (Figure 4).

Three broad themes describe adolescents' mental well‐being (Sarriera and Bedin 2017): psychological well‐being, subjective well‐being, and psychosocial well‐being. Younger adolescents were not able to express their opinion on adolescents' mental well‐being, while older adolescents gave clear opinions on this. In general, adolescents often mix two terms, mental health and mental illness. This conceptual confusion is prevalent among adolescents worldwide (Leighton 2009). Furthermore, older adolescents described adolescents' mental well‐being as quality of life, way of thinking and living (psychological well‐being), and a positive term which impacts their emotional well‐being (subjective well‐being). They stated that family and friends have an impact on their mental well‐being. Also, a big emphasis was given to the acceptance of others involved in interpersonal relations (psychosocial well‐being). Based on the study results, the following definition is proposed: ‘Mental well‐being is a positive term which describes a positive state of an individual where he/she accepts himself/herself, can function in society, and can exploit their potential’. Aspects that constitute mental well‐being are positive relationships with others, self‐acceptance, culturally supported values, and expectations. The mental well‐being of adolescents can be reached through interpersonal relations with others. Adolescents' understanding of mental well‐being differs due to their values, perceptions, expectations, and environment. Mental well‐being during adolescence is influenced by life experiences and relationships. Key protective factors include support by family members, a feeling of connectedness, supportive peers' help and support, and a supportive environment (World Health Organization 2016).

The category of interpersonal relationships is listed as an external category because interpersonal relationships affect the adolescent's experiences of mental well‐being and social support. It is evident that adolescents' perception of important people in their lives changes with their age. An interpersonal relationship is a relation between at least two people and should be based on support, trust, openness, communication, honesty, awareness of differences, equality, and respect. The family plays an important role in an adolescent's life, especially in early adolescence (starting at the age of 10). With their choices and lifestyle, devoted time and communication, they can influence adolescents' values and further relationships with others. This was proven in other studies (Betancourt et al. 2017; Moore et al. 2018; Stafford et al. 2016; World Health Organization 2016). Family influence is of great importance in an early phase of adolescence, whereas friends have greater importance in older adolescents' years (till 19 years of age). The importance of friends' support was also emphasised by (Engels et al. 2016). Ciarrochi et al. (2017) found that adolescents feel strong peer support and average to above‐average levels of well‐being. Adolescents' mental well‐being is also influenced by teachers' support. In a study including 1476 Chinese adolescents, authors reported that school‐related social support was correlated to adolescents' subjective well‐being (Tian et al. 2016).

Even though adolescents do not perceive registered nurses as very important people in their lives, they think that nurses have a lot of professional knowledge about mental well‐being (expert). Students stated that a nurse is someone they do not know well (foreign/stranger). However, it is easy to talk to him/her because he/she encourages them to engage in healthy activities (leader). Nurses give students useful information (source/resource) and teach them about a healthy lifestyle (teacher/health educator). Furthermore, students' opinions are that registered nurses support, care, help, advise (counsellor), and talk to adolescents (therapeutic role). Findings are in accordance with nurses' roles described by (Peplau 1952).

The focus on registered nurses rather than mental health physicians in this study is justified by their unique role in school settings. Unlike mental health physicians, who typically engage with students only during referrals or specific mental health interventions, registered nurses are consistently present within the school environment. This consistent presence allows them to establish ongoing relationships with adolescents, fostering trust and accessibility. Moreover, registered nurses often provide both preventive and educational interventions related to mental well‐being, which aligns with the holistic and continuous care model needed in school settings. Their ability to detect early signs of mental health issues and to offer initial support makes them essential in bridging the gap between students and specialised mental health care. Therefore, focusing on the role of registered nurses provides practical insights into enhancing school‐based mental health support.

### 4.1 Theoretical Implications

The mental well‐being of adolescents is poorly researched and studied. Also, there is no clear definition of the concept provided. A little attention is devoted to perceiving the mental well‐being of adolescents by parents, teachers, friends, registered nurses, and decision makers. The KIDSCREEN‐27 questionnaire has not been used in combination with the WEMWBS questionnaire. Both questionnaires showed good psychometrics and are valid for use among Slovenian adolescents. Study findings are of great importance and relevance for practice, education, research, and administration.

### 4.2 Practical Implications

Adolescents, family members, and carers must be involved in decisions about adolescents' care to provide high‐quality and person‐directed care, as adolescents perceive family members as important people in their lives. Moreover, as adolescents perceive registered nurses as important people in supporting their mental well‐being, it is important to involve nurses in schools to ensure holistic and continuous care for adolescents. Involving registered nurses in schools would provide proper mental health promotion, early recognition of mental health problems, early and appropriate actions, and support for adolescents and parents. Ongoing and coordinated healthcare for adolescents for periods after a mental health problem appears or is under control must be provided. Furthermore, various options for adolescents and parents must be provided to get the needed help when faced with mental health problems. Help should already be offered when a student enters the school and through the end of his/her education. Both healthcare and educational institutions must ensure professionals whom adolescents can talk to and who are able to help them if they struggle with poor interpersonal relationships, social exclusion, or other factors that may contribute to worsening mental well‐being and mental health.

### 4.3 Limitations and Future Research

Although the conducted study is well‐designed and involved a relatively large sample of adolescents, there are some limitations that should be taken into account when interpreting the study results. The conducted study is cross‐sectional, which does not allow us to explore the causal relationship between the phenomena. Moreover, only one repetition was used to determine the content validity of the WEMWBS scale. The stability of the scales using a test–retest correlation was not investigated. Some of the invited schools dropped out of the research; thus, new randomly selected schools were invited to participate in the study. A focus group of decision makers had limited participation due to different obligations. Also, there is a possibility that the scales did not fully cover the problem researched, but we estimate that with the added open‐ended question in the questionnaire, we were able to capture some additional information. The WEMWBS is a self‐reported questionnaire measuring mental well‐being in the past two weeks. The KIDSCREEN‐27 is a self‐reported questionnaire measuring social support, meaning that participants may give socially desirable answers. The study did not specifically assess whether participating students received formal mental health care or structured educational sessions from nurses. Although there are a few limitations listed, the authors believe that the findings of this study provide reliable and valid results that contribute new knowledge and understanding in relation to adolescents' mental well‐being and social support.

## 5 Conclusion

The mental well‐being of adolescents correlates with the support of the nursing and social environment, and the perception of this differs between adolescents, parents, teachers, registered nurses, and decision makers. Adolescents' mental well‐being differs between primary and secondary school students. Mental well‐being and social support drop with students' age. The most important people for primary school students are family members, and friends for secondary school students. Both primary and secondary school students agreed that good interpersonal relationships are based on trust, support, mutual respect, openness, honesty, awareness of differences, communication, understanding, and equality. Adolescents think that a nurse has an important role in maintaining adolescents' mental well‐being because he/she is an expert in the field of health promotion. Nevertheless, too little time is devoted to the discussion on mental well‐being. Nurses' role is to talk to adolescents, help them, and care for them. Nurses could have an important role in supporting adolescents when they have the necessary preparation, support, and the required commitment to partnership. Likewise, nurses are and should be welcome in schools to perform classes and workshops for adolescents, parents, and teachers. To ensure adolescents' mental well‐being, it is important that all factors and people possibly impacting an adolescent's mental well‐being are involved and taken into consideration.

### 5.1 Relevance for Clinical Practice

This study highlights the critical role of social support from family, friends, teachers, and registered nurses in promoting adolescents' mental well‐being. By showing how mental well‐being declines with age and correlates positively with social support, this research provides actionable insights for healthcare and educational professionals. Integrating registered nurses into schools for mental health promotion and early intervention can enhance support systems. This interdisciplinary approach equips practitioners with strategies to address adolescents' evolving mental health needs holistically.

## Author Contributions

**Leona Cilar Budler:** conceptualization, methodology, validation, formal analysis, investigation, writing – original draft, writing – reviewing and editing. **Majda Pajnkihar:** conceptualization, methodology, writing – reviewing and editing, supervision. **Gregor Stiglic:** conceptualization, methodology, validation, formal analysis, supervision. **Owen Barr:** conceptualization, methodology, writing – reviewing and editing, supervision.

## Funding

The authors have nothing to report.

## Ethics Statement

Ethical approval was also obtained from the Slovenian National Medical Ethics Committee (no. 0120‐313/2019/13).

## Conflicts of Interest

The authors declare no conflicts of interest.

## Data Availability

The data that support the findings of this study are available on request from the corresponding author. The data are not publicly available due to privacy or ethical restrictions.
